# Adaptive Vertical Positioning as Anti-Predator Behavior: The Case of a Prey Fish Cohabiting with Multiple Predatory Fish within Temperate Marine Algal Forests

**DOI:** 10.3390/ani12070826

**Published:** 2022-03-24

**Authors:** Pierre D. Thiriet, Antonio Di Franco, Adrien Cheminée, Luisa Mangialajo, Paolo Guidetti, Samuel Branthomme, Patrice Francour

**Affiliations:** 1 PatriNat (UAR OFB–CNRS–MNHN), Muséum National d’Histoire Naturelle—Station Marine de Dinard, 38 rue de Port-Blanc, 35801 Dinard, France; 2 UMR 7035 ECOSEAS, CNRS, Université Côte d’Azur, Parc Valrose, 28 Avenue Valrose, 06108 Nice, France; adrien.cheminee@septentrion-env.com (A.C.); luisa.passeron-mangialajo@unice.fr (L.M.); samuel.branthomme@unice.fr (S.B.); patrice.francour@unice.fr (P.F.); 3 Department of Integrative Marine Ecology, Stazione Zoologica Anton Dohrn, Sicily Marine Center, Lungomare Cristoforo Colombo (Complesso Roosevelt), 90142 Palermo, Italy; antonio.difranco@szn.it; 4 Septentrion Environnement, Campus Nature Provence, Lycée Professionnel Agricole des Calanques, 89 Traverse Parangon, 13008 Marseille, France; 5 Federative Research Institute MARRES, Université Côte d’Azur, CEDEX 2, 06107 Nice, France; 6 Department of Integrative Marine Ecology (EMI), Stazione Zoologica Anton Dohrn–National Institute of Marine Biology, Ecology and Biotechnology, Genoa Marine Centre, 16126 Genoa, Italy; paolo.guidetti@szn.it; 7 National Research Council, Institute for the Study of Anthropic Impact and Sustainability in the Marine Environment (CNR-IAS), 16149 Genoa, Italy

**Keywords:** habitat structural complexity, understory, canopy, forest, survival, habitat choice, anti-predator behavior, stalk-and-attack, sit-and-wait, ambush

## Abstract

**Simple Summary:**

Fish cohabiting within structurally complex habitats (e.g., coral reefs, seagrass meadows, algal forests) include abundant small-bodied prey fish and specialized piscivorous fish. Habitat structural complexity mediating fish predator–prey interactions has been shown to be an important mechanism sustaining this coexistence. However, the effect of the vertical stratification of habitat structure on predator–prey interactions remains poorly known, especially within a forest-like marine habitat, i.e., a habitat containing three vertical strata (understory, canopy and open-water). We set up tank experiments to test how such habitat vertical stratification affects predator–prey lethal and behavioral interactions, using one prey and two predator model species cohabiting in Mediterranean algal forest. We found that prey anti-predator behavior was predator-specific. When exposed to a sit-and-wait predator, the prey increased its vertical distance from the predator, regardless of the habitat structure. Conversely, when exposed to a stalk-and-attack predator, the prey sought refuge within forest structures. Prey hide motionless within the canopy, the most complex strata, while they avoid and escape from predators within the understory, which is a less complex stratum allowing for fast prey movements but still protected from predators by the canopy above. Our results suggest the crucial role of habitat vertical stratification in influencing predator–prey interactions, which should be studied in three dimensions.

**Abstract:**

Prey fish cohabit with specialized predator fish within structurally complex habitats. How the vertical stratification of the habitat affects lethal and behavioral predator–prey interactions and contributes to explaining these patterns has never been investigated within a forest-like marine habitat, i.e., a habitat containing three vertical strata (understory, canopy, open-water above). We studied this in tank experiments, with a model prey (the wrasse *Symphodus ocellatus*) and two model predators (the stalk-and-attack comber *Serranus cabrilla* and the sit-and-wait scorpionfish *Scorpaena porcus*), which are among the most abundant prey and predators cohabiting in Mediterranean *Cystoseira* forests. Wrasse anti-predator behavior was predator-specific. When exposed to the scorpionfish, the wrasse increased its vertical distance from the predator, regardless of the habitat structure. Conversely, when exposed to the comber, the wrasse sought refuge within forest structures: (1) the canopy provides more hiding opportunities due to its high complexity, and (2) the understory provides more escape/avoidance opportunities due to (a) its low complexity that allows for fast prey movements, and (b) the presence of the canopy above that limits the comber’s access to the understory. Our results suggest that habitat vertical stratification mediates predator–prey interactions and potentially promotes the co-existence of prey and multiple predators within marine forests.

## 1. Introduction

Habitats with high structural complexity (e.g., high substrate rugosity and/or high tree density) usually harbor high diversity and density of animals, in terrestrial [1], freshwater [2] and marine [3] ecosystems worldwide. Focusing on aquatic ecosystems, numerous small-bodied fish (small species and juveniles) are more abundant within vegetated habitats compared to adjacent unvegetated and less structurally complex habitats. Examples include seagrasses compared to adjacent bare sediments [4,5,6,7,8], mangrove roots compared to adjacent mud flats [9,10,11], giant kelp forests compared to adjacent bare rocks [12,13] and large brown algae *Cystoseira* forests compared to adjacent unforested habitats [14,15,16,17,18].

A reduction in predation pressure related to the greater availability of refuges has often been suggested as the main mechanism underlying the pattern of greater densities of small-bodied fish in complex vegetated habitats. This hypothesis is supported by numerous studies highlighting that vegetation provides prey fish with shelter and reduces predation-induced mortality [19,20,21,22]. However, exceptions can be found to this general pattern, depending on the species and systems considered. Some studies in fact have highlighted that some prey fish remain more abundant in vegetated habitats compared to adjacent bare habitats [6,7,22,23], despite the fact that those prey fish suffer from higher predation mortality in vegetated habitats hosting high densities of resident sit-and-wait predators (aka ambush predators), which are vegetation specialists [23]. This finding raises the question of how prey fish cohabit within vegetated habitats with abundant vegetation-specialist predators [24].

Predator–prey interactions within complex habitats remain poorly documented. Most prey fish anti-predator behavior has been studied in experimental choice arenas where the prey can choose between complex and open habitat, according to the presence and identity of predators [25,26,27,28]. However, natural habitats extend over larger surface areas than the small surface areas typically used in choice arena experiments. In nature, fish may have to make choices within the habitat rather than across two distinct habitats [29]. The few studies dealing with fish anti-predator behavior within predator-rich structured habitats (e.g., [29] in seagrass meadow; [30] in coral reef) have highlighted that the vertical positioning of prey was an important component of anti-predator behavior that was adapted as a function of the presence and identity of predators. In these studies, structured habitats were composed of only two vertical strata: the structured stratum (seagrass leaves or coral) and the open-water stratum above. To the best of our knowledge, predator–prey behavioral interactions have never been investigated within a forest-like marine habitat, i.e., a habitat containing three vertical strata: (1) the canopy stratum of high structural complexity (branches and ramifications of the canopy’s former macrophytes); (2) the understory stratum below, of lower structural complexity (main axis of the canopy’s former macrophytes); (3) the open-water stratum above. It is worth noting that the analogy to the “forest” relates to the vertical stratification [31], although the height of the canopy may be relatively low, as in the case of Mediterrannean *Cystoseira* forests that are usually 15 cm in height (see Figure S1).

To amend this lack of knowledge, and to better understand the process underlying the higher co-occurrence of certain prey and multiple predatory fishes in forest-like marine habitats compared to less structured adjacent habitats (notably less vertically stratified), the aim of the present study was to assess how the 3D structure of a forest may promote the anti-predator behavior of a prey fish facing two types of predators usually present in forest habitats: the stalk-and-attack predators and the sit-and-wait predators.

More specifically, using experimental tanks, we tested the following hypotheses:

**Hypothesis** **1** **(H1).** *Survival rate: prey survival is higher in forest-like habitat compared to shrub-like and barren habitats, whatever the type of predator;*

**Hypothesis** **2** **(H2).** *Habitat preference: prey that have the choice between forest-like and shrub-like habitats actively migrate into forest-like habitat, regardless of the absence or presence of and type of predator;*

**Hypothesis** **3** **(H3).** *Within habitat anti-predator behavior: prey escape/avoid predators mainly by adapting their vertical position in reaction to the position and identify of predators. Such anti-predator behavior is more effective in forest-like habitat due to the higher vertical stratification.*

## 2. Materials and Methods

### 2.1. Model Species and Habitats

We used as model species (1) the small-bodied wrasse *Symphodus ocellatus* (hereafter ‘wrasse’) as model prey, and (2) separately, two model predators characterized by distinct foraging strategies: the stalk-and-attack *Serranus cabrilla* (hereafter ‘comber’) and the sit-and-wait *Scorpaena porcus* (hereafter ‘scorpionfish’). These two species are among the most important predators of small-bodied fish in the north-western Mediterranean Sea, although they are opportunistic macro-carnivores that also feed on crustaceans [32,33].

These three species were used since they are among the most abundant prey and predators cohabiting in Mediterranean *Cystoseira* forests, a complex habitat composed of 3 vertical strata: the canopy formed by *Cystoseira* spp., the understory below and the open water above [34]. Moreover, the 3 model fish species are less abundant in alternative less-complex habitats such as shrub-like habitats (Dictyotales- and Sphacelariales-dominated assemblages, hereafter called ‘shrub’) and barren habitats (bare rocks with coralline algae, hereafter called ‘barren’) [14,15,16,17] (Figure S1 details the 3D structure of these 3 habitat types).

### 2.2. Fish Collection and Housing

All the individuals used for the tank experiments were collected by scuba-diving on shallow rocky reefs at Villefranche-sur-Mer Bay, French Riviera (43°41′42.77″ N, 7°18′28.10″ E) between 18 April and 1 July 2011. Fish individuals were first selected by eye in order to have fish as homogeneously sized as possible, and then measured (TL) to the nearest mm. Wrasse [mean (SD) TL: 46 (2.7) mm; n = 60] and scorpionfish [138 (9.6) mm; n = 32] were caught using hand nets, while comber [130 (4.6) mm; n = 44] were fished using lines and hooks. Each fish was used for only one trial to ensure independence among replicates (see Section 2.5 Statistical analyses).

After collection, fish were held separately by species in tanks. Holding tanks (capacity between 340 and 620 L) and experimental tanks (see Section 2.3) were all placed in the same room but were isolated from each other using dividing walls so that no visual interference could affect behavioral observations. Each tank had neon lights 1.5 m above, turned on for 16 h/day. Holding and experimental tanks were filled with synthetic sea water (salinity: 37 (+/−0.5) p.p.m.; temperature nearly constant at 21 °C). Water treatment of each tank was an individual closed-loop system oxygenated with a water pump and filtered through a polyamide membrane. Treatment systems of experimental tanks also included an activated-carbon filter (see Section 2.3). Physical–chemical parameters pH, KH, Ca, NO_2_ and NO_3_ were checked using 5-in-1 test strips, and water was renewed when necessary. Fish were fed every day: wrasse with defrosted brine shrimp *Artemia salina* and predators with defrosted mussels *Mytilus galloprovincialis*. Before using individuals for the experiments, they were held for 14–30 days in holding tanks, to allow for recovery and acclimation to tanks and artificial algae [35]. Predator fish as well as prey fish were not fed for 24 h before the experiment started, to make sure that they started from the same hunger status among replicates, and to avoid variability due to the putative effect of hunger status on both prey and predator behavior.

### 2.3. Experimental Design and Procedures

**Figure 1:**
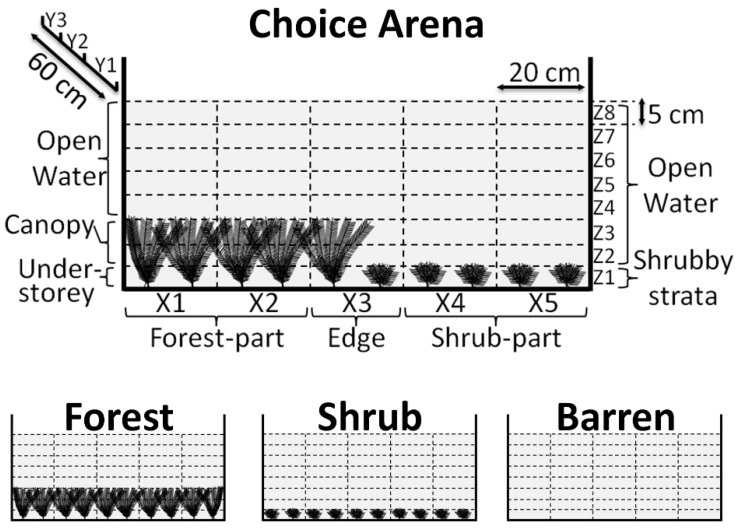
Schematic representation of the 3 artificial habitat types and the choice arena. Tanks were 100 × 60 × 40 cm and virtually divided by a 3D grid (X, Y and Z axis) for recording fish positions. Stems of plastic algae were used for mimicking vegetation (see also Figure S3 showing pictures of artificial set up).

Four identical 100 × 60 × 40 cm tanks were used for the trials. Four distinct types of artificial habitats were created: barren (B), shrub (S) and forest (F), plus a choice arena (CA). The latter was specifically dedicated to testing a putative habitat preference for forest over shrub habitat. It was composed of 50% shrub and 50% forest (Figure 1 and Figure S3). The bottom of each artificial habitat consisted of a green velour carpet. In S and F habitats, green plastic algae were added to the carpet, one plant every 10 cm (100 individuals m^−2^). Each artificial algae plant (model P13EL of PENN-PLAX as raw material) was made with five stems 5 cm-long for shrub habitat, and five stems 15 cm-long for forest habitat. This setting was chosen on the basis of a preliminary field survey and previous work [14]. Artificial habitats were removable so that before every trial, the 4 distinct habitats were randomly set up within the 4 experimental tanks. The choice arena was randomly oriented within its tank (i.e., forest on the left or the right).

For each habitat, 5 distinct treatments involving prey and/or predator were performed (hereafter referred to as predator–prey treatments). Three treatments, used as controls (see Section 2.4), involved one individual alone: one wrasse (W), one scorpionfish (S) and one comber (C). The two other treatments involved one predator and one prey together: one scorpionfish and one wrasse (SW), and one comber and one wrasse (CW).

Before every trial, each experimental tank remained empty of fish and its closed-loop water-treatment system was turned on for 24 h. The treatment systems included an activated-carbon filter to reduce concentrations of dissolved fish chemical cues from the previous trial. We assume this allowed the concentrations to reach trace levels, and considering that such traces are ubiquitous in the field, we assumed these could not significantly affect the next trial [26]. Moreover, in the unlikely case that chemical cues from the previous trial might affect the next trial, the effects would be additional noise rather than directional bias because of the randomization of the order of treatments and their individual assignment to one or other of the 4 experimental tanks. After the 24 h of water treatment, one hour before starting data collection, water-treatment systems were turned off and prey and/or predator were introduced into the experimental tank. This hour enabled fish to get over the stress caused by manipulation, and to explore and become accustomed to the experimental tank. For treatments including both prey and predator, individuals were isolated from each other during the 1h adaptation period by an opaque plastic plank. Within the choice arena, both prey and predator had access to the two habitats. Trials started as soon as the opaque separation planks were removed. The water-treatment systems remained turned off throughout the behavioral and survival experiments in order to avoid noise and currents that could potentially affect fish behavior, including positioning (e.g., fish reacts to the local stream generated by the pump) that is an important response variable in habitat choice and micro-habitat use experiments (see Section 2.5).

Every combination of habitat and predator–prey treatment was replicated 4 times (i.e., 4 trials), excluding the combinations involving the predator–prey treatment CW, which were replicated 7 times. Higher replication for CW was due to the short survival time of prey when exposed to comber (see Section 3.1), and the aim was to collect enough behavioral data. Every individual was exclusively used for a single trial in order to avoid non-independent observations.

### 2.4. Survival and Behavioral Data Collection

All observations were diurnal (artificial neon light) due to logistical constraints (i.e., the need to visually assess fish position, behavior and survival). It was not possible to observe and/or record encounter, attack and capture rates, because wrasse individuals in the forest were not permanently visible. On the basis of 3 preliminary trials where wrasse never stayed unseen longer than 30 s, a multiple-snapshot sampling strategy was adopted. Survival and behavioral data for prey and/or predator were collected on 63 occasions during each trial. Each trial lasted 130 min, and data were collected on 21 occasions (every 30 s) per each 3 observational sessions which were 10 min long—S1: from 0 to 10 min; S2: from 60 to 70 min; S3: from 120 to 130 min. For CW treatments, predation events interrupted observations; thus, the total number of behavioral observations per trial was less than 63. For SW treatments, prey survived the 130-min trials and data on survival were collected until prey disappeared due to predation.

Fish individuals’ positions were recorded using semi-quantitative variables. Tanks were virtually divided into a 3D grid. The horizontal axes X and Y were split every 20 cm and the vertical axis Z was split every 5 cm. Higher spatial resolution on the Z axis was related to the specific focus on prey vertical movements. It is worth noting that Z values do not match the same micro-habitats according to the habitat type (Figure 1). In order to estimate predator–prey distance, categorical positions were transformed into numerical coordinates (by taking the mid-point of the intervals) and Euclidean distances were used.

Predator activity was classified into two categories: mobile vs. motionless [27]. Prey activity was classified in three categories: mobile, exposed motionless, and hidden motionless. The latter category was used when the prey positioned its body against a stem of algae.

The observer collected data by moving discretely all around the tank as often as necessary. The observer’s body was hidden behind a 120 cm high wall that surrounded the tank at a distance of 60 cm from it (see Figure S2). We assume this minimized interference affecting fish behavior. Furthermore, we assume any possible remaining interference could not affect the main results of our comparisons between treatments, since the interference would have equally affected all treatments.

### 2.5. Statistical Analysis

#### 2.5.1. Effects of Habitat Structure on Lethal Interactions

Prey survival (or predator foraging efficiency, depending on the perspective) was analyzed separately for the two predators due to the different number of replicates and difference in time scales (hours vs. days, see Section 3.1). To compare prey survival curves (i.e., the percentage of prey alive over time) between habitats, the class of non-parametric maximum likelihood estimators of the survival functions were fitted. This enabled us to properly deal with the non-uniqueness of survival functions inherent in the interval-censoring nature of the data [36]. The test for equality of survival functions between habitats was carried out using Asymptotic Log-rank (Sun’s scores) k-sample test. When the equality hypothesis was rejected, pair-wise comparisons between pairs of habitats were performed using asymptotic log-rank 2-sample test on data subsets and Holm-corrected *p*-values. All the survival analyses were implemented with the ‘interval’ package [36] of the R statistical and programming environment [37].

#### 2.5.2. Statistical Unit and General Method Used for Comparing Averaged Behavior between Habitats

When comparing prey or predator behaviors between habitats and predator–prey treatments, behavioral observations were aggregated at the individual level in order to obtain+ independent statistical units. To obtain a representative average behavior, only trials including at least 21 observations (i.e., trials with the prey alive after the first observational session ended) were used. This excluded all CW trials within barren habitat, 1 CW trial within shrub habitat and 1 within forest (see Section 3.1). All univariate and multivariate permutational analyses of variance (PERMANOVA) and subsequent pair-wise tests were conducted with the software PRIMER 6/PERMANOVA+, using Euclidean distance and 9999 permutations under the reduced model. Marginal sums of square (type III) were used since designs were unbalanced and some cells were empty. Monte-Carlo p-values were considered when not enough permutations were possible (i.e., <200) [38]. In addition, 95% confidence intervals of all reported mean values were estimated using 9999 bootstrap re-sampling of fish individuals.

#### 2.5.3. Effects of Predator–Prey Co-Occurrence on Their Respective Habitat Selection

Habitat selection (choice between forest and shrub habitat) was investigated for each species under each predator–prey treatment (the species alone or with prey/predator). The Jacob’s D Selection Index (SI, [39]) was compared between the choice arena (half forest, half shrub, see Figure 1) and the two homogenous habitats (Forest and Shrub), which served as controls for artifact. In control habitats, selection for a particular side of the tank might indicate artifact. SI was computed for each individual fish (the independent sampling unit), as follows:

$$SI=\frac{n_{S}-n_{F}}{n_{S}+n_{F}},$$

where *n* represents the number of times the individual was observed in the forest part ($n_{F}$, i.e., X1 and X2 pooled) and shrub part ($n_{S}$, i.e., X4 and X5 pooled) of the choice arena (Figure 1). In the two control habitats shrub and forest, $n_{F}$ and $n_{S}$ matched the orientation of the respective parts of the choice arena (that was randomly oriented prior to every trial). The edge part (X3) was excluded to avoid potential confusion between habitat selection and possible edge effect.

SI ranges between −1 and 1. SI = 0 means no selection. SI_choice-arena_ = −1 means perfect selection of forest part over shrub part of the choice arena. SI_choice-arena_ = 1 means perfect selection of shrub part over forest part of the choice arena. SI_forest_ and SI_shrub_ aimed at controlling for artifacts. Their values are expected to be 0 on average (no selection). SI_forest_ = −1 means perfect selection of the part of the control tank that was oriented in the same direction as the forest part of the choice arena. SI_forest_ = 1 means perfect selection of the part of the control tank that was oriented in the opposite direction to the forest part of the choice arena. SI_shrub_ interpretation is similar to SI_forest_. Hence, habitat selection holds if on average SI_shrub_ = SI_forest_ = 0 ≠ SI_choice-arena_. For each species, putative differences in mean SIs between habitats (S, F, CA) and predator–prey treatments (alone or with prey/predator) were tested using 2-way crossed (univariate) permutational analysis of variance (PERMANOVA). Pairwise comparisons were performed whenever necessary. Signs of mean SI values (<0, =0 or >0) were assigned using their 95% confidence intervals.

#### 2.5.4. Effects of Habitat Structure on Averaged Behavioral Interactions

Fish individuals’ average behavior (in terms of vertical position, activity level and predator–prey distance) was compared between habitats and predator–prey treatments. Distributions of vertical position (Z) and predator–prey distance (PPD) consisted of 21 to 63 semi-quantitative measures per individual. In order to aggregate distributions at the level of the individual (the independent sampling units), the mean and the standard deviation of each individual fish’s distribution was used. The standard deviation (hereafter referred to as “variation”) may be seen as a proxy of the preference strength for the mean value. Four aggregated variables were obtained: mean vertical position, vertical variation (around the mean), mean PPD and PPD variation. Mean of each variable was compared between habitats (B, S, F) and predator–prey treatments using univariate PERMANOVA. The categorical variable ‘activity’ was expressed for each individual as frequencies per category. Frequencies were organized in a matrix (fish individual X category) and were compared between habitats and predator–prey treatments using multivariate PERMANOVA.

## 3. Results

### 3.1. Effects of Habitat Structure on Lethal Interactions

**Figure 2:**
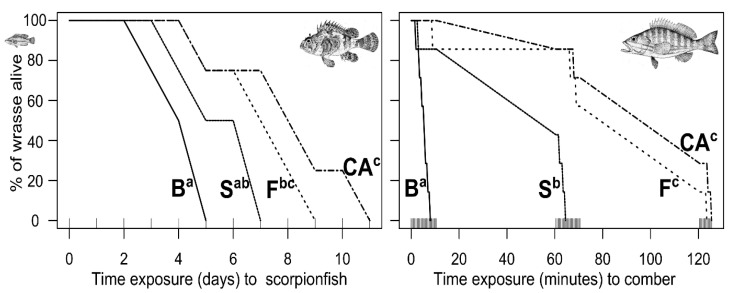
Survival curves of wrasse depending on habitat types and predator identity. Wrasse were exposed to scorpionfish (**left**) and to comber (**right**) in the habitats of increasing complexity: Barren (B), Shrub (S) and Forest (F), and in the Choice Arena (CA). In each graph, curves sharing a lowercase letter were not significantly different. Upper tick marks on the axis X delimits the censored intervals.

Predators’ foraging success was reduced by increasing habitat complexity. Wrasse survival curves were significantly different between habitats when wrasse were exposed both to scorpionfish (Log-rank test, X^2^ = 11.8, df = 3, *p*-value = 0.008) and comber (Log-rank test, X^2^ = 40.3, df = 3, *p*-value < 0.001). In both cases, survival curves were steeper in barren than in forest and choice arenas (half shrub–half forest), and intermediate in shrub (Figure 2). Although prey survival curves were not statistically compared between predators, comber preyed faster than scorpionfish. All predation events occurred within 130 min in the arenas with comber, while predation events occurred only after 3 days minimum in the arenas including scorpionfish. For the 7 replicates of the wrasse–comber treatment in barren habitat, wrasse were preyed on before the first observational session ended (i.e., <10 min, Figure 2).

### 3.2. Effects of Predator–Prey Co-Occurrence on Their Respective Habitat Selection

**Figure 3:**
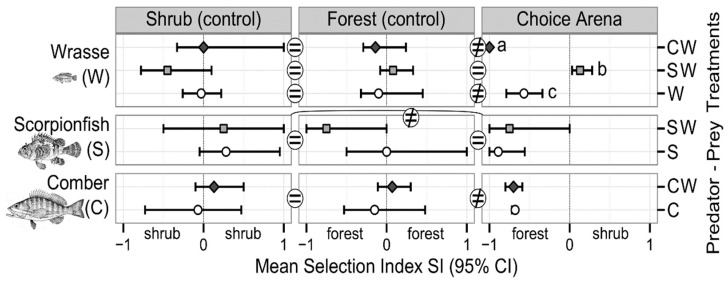
Habitat selection of prey and predators depending on predator–prey treatments. Selection Index (SI) mean values (95% CI) are presented in controls (shrub and forest, left and middle panel columns) and choice arena (right panel column), for wrasse (upper panel row), scorpionfish (middle panel row) and comber (lower panel row), when they are alone (predator–prey treatment W, S or C labelled on the right) or when prey and predator are together (SW or CW labelled on the right). In choice arena, SI < 0 means selection of forest part over shrub part, SI > 0 means the opposite. Results of post hoc pair-wise comparisons post hoc to ANOVA (Table 1) are reported using equal/unequal symbols or lowercase letters. Habitat selection holds if on average SI_shrub_ = SI_forest_ = 0 ≠ SI_choice-arena_. See Section 2 for more details.

Wrasse habitat selection was related to predator presence and identity (Figure 3, Table 1). Wrasse preferred the forest over the shrub habitat when they were alone or in the presence of comber, while no habitat selection was observed in the presence of scorpionfish. More specifically, for both treatments W and CW, pair-wise comparisons of SIs (Figure 3) revealed that mean SIs differed between controls and choice arena, being close to 0 in controls and negative in the choice arena (i.e., mean SI_shrub_ = mean SI_forest_ = 0 > mean SI_choice arena_). Moreover, mean SI_choice arena_ was more negative for W than for CW treatment. This may indicate that the presence of comber strengthens wrasse’s preference for forest. In contrast, when wrasse were in the presence of scorpionfish, they did not select any habitat, since mean SIs did not differ between habitats and were close to 0.

Comber habitat selection was independent of wrasse absence/presence (Table 1). Comber preferred the forest over the shrub habitat. More specifically, comber SIs were significantly different between habitats and pair-wise comparisons (Figure 3), revealing that mean SIs differed between controls and choice arena (i.e., mean SI_shrub_ = mean SI_forest_ ≠ mean SI_choice arena_), being close to 0 in controls (confidence intervals overlapping 0) and negative in the choice arena.

### 3.3. Effects of Habitat Structure on Averaged Behavioral Interactions

#### 3.3.1. Activities

The wrasses’ activity was different between predator–prey treatments (Table 2). When alone and in the presence of scorpionfish, wrasse were mobile during 81% (mean; 95% CI: 77, 84) of the observations and were exposed motionless during all the other observations. In contrast, in the presence of comber, wrasse were (only in shrub and forest habitats, see Section 2) mobile during 21% (mean; 95% CI: 17, 26) of the observations, exposed motionless during 4% (mean; 95%CI: 2, 6), and hidden motionless during 75% (mean; 95% CI: 71, 79).

Scorpionfish were observed motionless most of the time, but some slight differences were detected (Table 2) between the absence (97% (90, 99)) and the presence of wrasse (100% (98, 100)), and between habitats: motionless 94% (88, 98) of the time in barren habitat, 99% of the time (94, 100) in shrub habitat, and 100% of the time (99, 100) in forest.

The combers’ activity was dependent on the presence of wrasse (Table 2). Comber were observed mobile and motionless in 52% (50, 55) and 48% (43, 51) of the trials when they were alone, respectively, while their mobility increased to 71% (69, 76) in the presence of wrasse.

#### 3.3.2. Vertical Distribution

**Figure 4:**
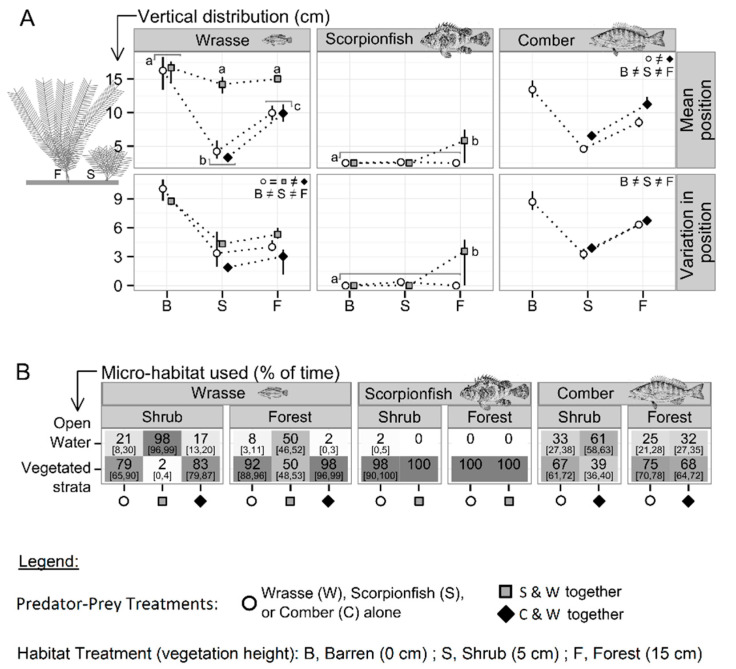
Vertical distributions of prey and predators depending on vegetation height and predator–prey treatments: (**A**) values averaged over replicates [95% CI] of the means (Mean position) and the SDs (Variation in position) of every individual’s vertical distributions. Results of pair-wise comparisons post hoc to ANOVA (Table 3) are reported using equal/unequal symbols or lowercase letters. (**B**) Mean frequencies [95% CI] of the time spent within the vegetated strata (0 to 5 cm in Shrub, 0 to 15 cm in Forest) and within open water. The intensity of shading is proportional to mean values.

Wrasse adapted their vertical distribution in response to the interaction of habitat and predator–prey treatments (Figure 4A, Table 3), with major changes (relative to wrasse alone) taking place in reaction to the presence of scorpionfish and minor changes in reaction to the presence of comber.

Wrasse in the absence of predators adapted their vertical distribution in response to the vegetation height (Figure 4A and Table 3). In barren habitat, wrasse did not prefer any specific vertical stratum. Mean positions corresponded to 15 cm (around the middle of the water column) and position variations were high (Figure 4A). In contrast, in the vegetated habitats, vegetated strata were preferred. Position variation was lower, involving an increased preference for mean position, which was also lower. Mean position was higher in forest than in shrub habitat (Figure 4A), but the greater vegetation height in forest compared to shrub habitat enabled wrasse to more frequently occupy the vegetated strata in forest than in shrub habitat: in forest, 92% (mean; 95% CI: 88, 96) of the observations were within vegetated strata (understory and canopy); in shrub habitat, 79% (mean; 95% CI: 65, 90) of the observations were within shrub stratum (Figure 4B). Nevertheless, in both vegetated habitats, wrasse in the absence of predators clearly preferred the vegetated strata over open water.

Wrasse radically changed their vertical distribution when they were exposed to scorpionfish in shrub habitat and in forest. Preference for vegetated strata was replaced by preference for intermediate distance from the bottom, regardless of the habitat. Mean positions were in both habitats around 15 cm above the bottom, with moderate position variations (Figure 4A), which led to distinct frequencies of vegetated strata occupation (Figure 4B). In barren habitat, mean position was also around 15 cm above the bottom but position variation was higher, as for wrasse in the absence of predators (Figure 4A).

Wrasse changed their vertical distribution slightly when exposed to comber. Mean position and percentage of time spent within vegetated strata did not change (Figure 4B); only position variation was reduced (Figure 4A).

Scorpionfish’s vertical distributions responded significantly to the interaction between habitat and predator–prey treatments (Table 3 and Figure 4A), all being identical except for scorpionfish in forest with wrasse. Except for the latter case, scorpionfish were almost always observed positioned motionless on the bottom. When in forest in the presence of wrasse, scorpionfish were observed in 33% (mean; 95% CI: 8, 50) of cases positioned motionless in the upper-most part of the canopy, and in 67% (mean; 95% CI: 42, 83) of cases positioned motionless on the bottom below the canopy.

Comber adapted their vertical distribution in response to habitat treatments (in terms of mean position and position variation) and in response to prey presence (in terms of mean position) (Table 3 and Figure 4A).

Comber alone adapted their vertical distribution in response to the vegetation height (Figure 4A and Table 3). In barren habitat, comber did not prefer a specific vertical stratum, since mean position corresponded to the middle of the water column and position variation was high (Figure 4A). In contrast, position variation was intermediate in forest and low in shrub (Figure 4A), meaning that preference for mean position increased in the vegetated habitats. Mean position was higher in forest than in shrub (Figure 4A), but canopy and shrubby strata were similarly frequented. Comber alone in shrub habitat was observed in 67% (mean; 95% CI: 61, 72) of cases within shrubby stratum, and comber alone in forest habitat was observed in 75% (mean; 95% CI: 70, 78) of cases within vegetated strata (Figure 4B). Hence, comber in the absence of wrasse preferred vegetated strata over open water, in both vegetated habitats.

Comber adapted their vertical distribution in response to the presence of wrasse, with an increase in their mean positions (Figure 4A). In shrub habitat, this resulted in a decrease in the time spent in the shrubby strata (39% (mean; 95% CI: 36, 40)). In forest, the time spent within the whole of the vegetated strata did not significantly decrease (68% (mean; 95% CI: 64, 72), Figure 4B).

#### 3.3.3. Predator–Prey Distances

Distributions of predator–prey distance (PPdist) were different between habitat treatments in terms of mean (2 ways PERMANOVA, only term Ha significant: Pseudo-F_3,28_ = 13.37, *p* < 0.001), and were different between predator–prey treatments in terms of variation (2 ways PERMANOVA, only term Pr significant: Pseudo-F_1,28_ = 15.76, *p* < 0.001). Post hoc tests revealed that mean PPdist were highest in barren habitat and shrub habitat, intermediate in forest and lowest in the choice arena. PPdist variation was higher with comber than with scorpionfish (Figure S4).

### 3.4. Within-Habitat Description of Short-Term Behavioral Interactions

**Figure 5:**
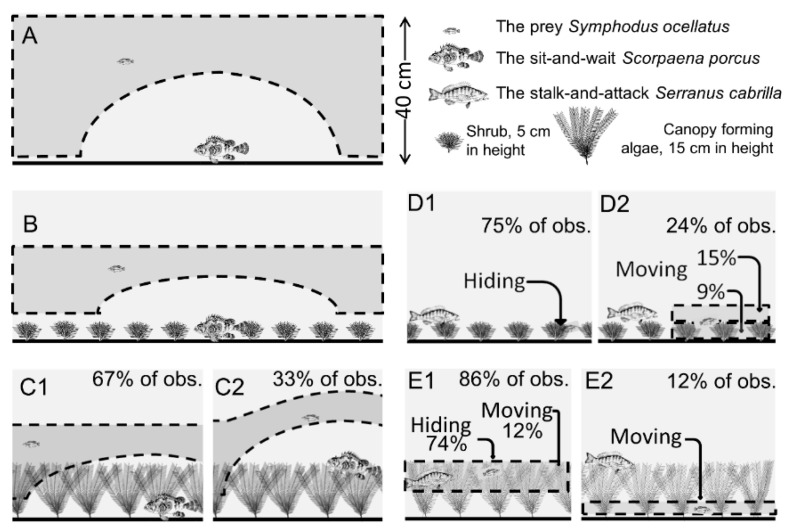
Schematic representations of the predator-specific anti-predator behavior of wrasse, depending on habitat types. Wrasse and scorpionfish behavioral interactions within bare control habitat (**A**), within shrub habitat (**B**) and within forest habitat (patterns **C1** and **C2** observed in 67% and 33% of cases respectively); wrasse and comber behavioral interactions within shrub habitat (patterns **D1** and **D2** observed in 75% and 25% of cases respectively) and within forest habitat (patterns **E1** and **E2** observed in 86% and 12% of case respectively). Formal tests of associations among categories of activity, prey and predator positions and predator–prey distances are reported in Supplementary File S1.

Besides the analysis of the effects of habitat structure on the averaged behavioral interactions reported above, we also quantitatively analyzed within-habitat short-term behavioral interactions. Statistical methods and detailed results are provided in the Supplementary File S1. In brief, Figure 5 schematically represents how prey and predator activity, vertical positioning and distance from each other interact (with statistical significance) within each habitat.

Within barren habitat, wrasse in the presence of scorpionfish (Figure 5A) moved across every stratum of the water column but wrasse’s movements were constrained by the positions of the scorpionfish. When the wrasse was swimming above the scorpionfish positioned motionless on the substrate, the wrasse increased its vertical distance from the substrate. Wrasse used the same tactic in reaction to the scorpionfish within shrub habitat and forest, with some slight variations. Wrasse did not swim close to the surface nor to the substrate in both vegetated habitats (Figure 5B,C1), except when the scorpionfish was positioned motionless on the canopy of the forest (Figure 5C2). In the latter case, the wrasse reacted by increasing its distance from the substrate. Hence, the wrasse always adapted its vertical position to avoid immediate proximity to scorpionfish.

Wrasse behaved differently when exposed to comber. In both vegetated habitats, wrasse remained hidden within the vegetated strata for most of the time (Figure 5D1,E1). Wrasse’s movements differed between habitats. In shrub habitat, wrasse mainly moved in open water just above the shrubby stratum, and to a lesser extent within the shrubby stratum (Figure 5D2). In forest, wrasse always moved within the vegetated strata (Figure 5E2). With regards to the use of the different vegetated strata of the forest, wrasse always hid within the canopy, and moved within the canopy and within the understory (Figure 5E1,2).

## 4. Discussion

Fishes are more abundant and their assemblages are more diverse within structured habitats [4]. These patterns are shared by numerous fish species, including both predators and prey, that often cohabit within the same habitats [24]. However, predator–prey behavioral interactions within structured habitats and the role of habitat structure are not well-known. Our tank experiments, by using artificial algae, highlighted the paramount effect of habitat structural complexity on active habitat selection by both predatory and prey fish, as well as on foraging success of predators and survival of prey. The wrasse *Symphodus ocellatus* has an adaptive anti-predator behavior pattern related to habitat structure when exposed to the stalk-and-attack comber *Serranus cabrilla*, since wrasse seeks refuge within vegetation stratae. In contrast, when exposed to the sit-and-wait scorpionfish *Scorpaena porcus*, the adaptive anti-predator behavior of wrasse is not affected by habitat structure, since wrasse simply increase their vertical distance to the dangerous bottom where the scorpionfish sit. Our results suggest that habitat vertical stratification and prey adaptive vertical movements may contribute to the co-existence of prey and multiple predators within structured habitats.

### 4.1. The Paramount Effect of Habitat Structure on Fish Habitat Selection

Artificial algae were used in our tank experiments in order to assess the putative effects of habitat structure in itself, free from any confusion related to other factors such as food (normally associated with natural algae). With regards to the behavior patterns of the three species observed individually, wrasse and comber adapted their vertical distribution in response to vegetation height. Moreover, wrasse and comber preferred the forested structure over the shrubby structure. These observations suggest that habitat structure is an intrinsically important factor affecting fish decision making regarding both vertical distribution and habitat selection. Concerning behavior patterns when prey and predator are together, predation risk (foraging opportunity, alternatively) seems to be less important than habitat structure in influencing the decision making of prey (predator, alternatively) regarding habitat selection. Wrasse exposed to comber in the choice arena still chose the forest-part. This occurred despite the immediate vicinity of the comber, which also chose the forest part. Wrasse’s preference for the predator-rich habitat of the forest might be related to the fact that prey may escape predation because of the forest canopy (see Section 4.2), which may compensate for the higher encounter rates with predators [40]. Comber’s preference for forest, on the other hand, does not necessarily reduce its foraging efficiency in the field. Forests host higher densities of comber’s favored prey (small fish and macroinvertebrates) [32,41]. This may give the comber more opportunities to attack, which may override the lower attack success rate and ensure an overall higher foraging success rate [42]. Additionally, considering that comber may possibly be prey of larger roving predators (e.g., *Dentex dentex*), its preference for forest may also be related to an anti-predator behavior. Wrasse faced with scorpionfish did not select any specific part of the choice arena. This lack of selection probably resulted from two conflicting demands: wrasse’s preference for forested structure and the avoidance of scorpionfish, which was always positioned in the forest part of the choice arena. In the field, wrasse may likely solve this conflict by moving a few meters away, but still remaining within the forest.

Habitat selection could be primarily due to habitat preference, related only to the intrinsic preference for a given habitat structure and not triggered by predation risk or food availability [43,44]. However, habitat preference may be a proactive behavior, possibly learned and/or selected through predation and/or foraging pressure [43,44,45,46,47]. Experiments including all combinations of food availability, predation risk and habitat structure are required to assess the most important proximate cues used by fish for selecting habitat (e.g., [43]). Nevertheless, our tank experiments suggest that habitat selection (and perhaps primarily habitat preference) may induce net migration of fish into forest and contribute to shaping the density patterns observed in the field, with high fish diversity and abundances observed in *Cystoseira* forests [14,15,16,17].

### 4.2. Comber–Wrasse Within-Habitat Behavioral Interactions

Comber employed an active-search foraging strategy in every habitat. Comber increased their mobility in response to the presence of wrasse and they explored the same micro-habitat as wrasse (the respective vertical distributions of comber and wrasse matched in every habitat). This observed behavior coincides with the well-known diurnal stalk-and-attack comber foraging strategy [48,49]. When exposed to comber, wrasse reduced their mobility and hid within vegetation when available (i.e., in shrub habitat and forest habitat), with a clear preference for hiding in forest over shrub habitat. The absence of a vegetated stratum in barren habitat did not allow the wrasse any opportunity to hide. This explains the lower survival rates observed in barren habitat. Wrasse’s preference for forest over shrub habitat and its higher survival rate in forest compared to shrub were probably due to the higher habitat complexity of forest, but perhaps more specifically to its vertical stratification. In forest habitat, wrasse most often used the denser upper part of the canopy (branches and leaves) for hiding when predators were close. This was probably because high structural complexity limits visual cues for predators and/or reduces predator mobility [50,51]. On fewer occasions, wrasse were observed to move. In the forest, wrasse moved within the canopy and also within the less complex understory (trunks, mains axis), especially when comber were up near the canopy. This tactic seems to rely on the particular stratification of the forest: an understory suitable for fast prey movement that is moreover well-protected by the canopy above that acts as a horizontal barrier limiting predator access to the understory. In contrast, in the shrub habitat, wrasse moved by passing among shrubs and also by passing above the shrubby strata. This latter tactic might be adopted to widen the field of view in order to better assess predation risk [52]. While doing so, wrasse are not hidden and are detected more easily by comber. We suggest that forest, compared to shrub habitat, increases the efficiency of wrasse anti-comber behavior by providing more hiding opportunities because of the overall higher structural complexity (canopy vs. shrubby strata) and/or because of the vertical stratification that provides more escape/avoidance opportunities due to the presence of an understory, the low complexity of which allows for fast prey escape while the canopy above limits comber’s access.

### 4.3. Scorpionfish–Wrasse Within-Habitat Behavioral Interactions

Wrasse’s anti-scorpionfish behavior consisted of avoidance, and this did not depend on the habitat structure. Wrasse did not select either of the two habitats during our habitat-choice experiment, and on the other hand, wrasse behaved similarly within each habitat. Within each habitat, wrasse moved and increased their vertical position when passing above the predator in order to avoid its immediate vicinity. This avoidance tactic was efficient since wrasse survived for at least 3 days. This efficiency is probably due to the scorpionfish sit-and-wait strategy that can succeed only if the prey comes within its limited attack range [53]. In the field, where fish are not confined, the avoidance tactic we observed probably causes wrasse to move a few meters away from the sit-and-wait predator. From this perspective, increasing vertical distance from the predator (hereafter referred as ‘vertical avoidance’) may be the initial response to predator detection, followed by horizontal avoidance (within or across habitats). Horizontal avoidance of sit-and-wait predators is well-known, especially in the context of habitat selection. Numerous studies in freshwater [25,26] and marine systems [6,28] have reported such behavior, where prey avoid a sit-and-wait predator positioned in the vegetated habitat by shifting to the adjacent predator-free non-vegetated habitat. In contrast, the only example of vertical avoidance concerning bentho–pelagic systems is the y-0 cod (*Gadus morhua*) against the sit-and-wait sculpin *Myoxocephalus scorpinus* [29]. Early vertical avoidance of wrasse exposed to sit-and-wait predators within stratified habitats may be related to the fact that detection of predators hidden within the complex stratum may be olfactory before being visual [26]. A prey olfactorily detecting the presence of a sit-and-wait predator but that does not exactly locate it (visually) may have an advantage in avoiding the dangerous complex stratum by directly going up into the open-water strata. However, our behavioral evidence for scorpionfish should be taken with caution considering that, due to logistical constraints, all behavioral observations were conducted under artificial light during the day phase of the day/night cycle, while scorpionfish are known to be more active at night.

### 4.4. The Apparent Low Foraging Efficiency of Scorpionfish

Scorpionfish needed at least 3 days (and nights) to capture wrasse. This apparently low foraging efficiency may be due to multiple processes. (1) Scorpionfish may have not been hungry during the first days, because of the low energy cost of the sit-and-wait strategy [53] and/or due to post-manipulation stress (transfer from holding to experimental tank) and/or observer presence during observations. (2) Scorpionfish might have waited during the first days, expecting other easier to catch prey such as brachyurans [54]. Finally, (3) scorpionfish might have tried but failed to capture wrasse during the first days and nights. Considering that some stomach content analyses have revealed that scorpionfish are able to capture bentho-pelagic fish in large quantities, such as the red mullet *Mullus barbatus* [55] and even pelagic fish such as the European anchovy *Engraulis encrasicolus* [56], the apparent low foraging efficiency might be due to wrasse’s anti-scorpionfish behavior that may have been particularly efficient, at least during the first days and nights. Nevertheless, we cannot exclude that our artificial habitats were not realistic enough and may have affected negatively the scorpionfish. The lack of crevices, rocks and different macrolagal assemblages might have prevented scorpionfish from hiding efficiently, as camouflage is important in the sit-and-wait strategy. Nevertheless, after some days, scorpionfish preyed on wrasse more efficiently in barren habitat than in forest. This was unexpected, because the sit-and-wait foraging strategy is recognized to be unaffected by structural complexity (e.g., [7]). Other studies even suggest that habitat complexity enhances its efficiency (e.g., [57]), possibly by promoting predator camouflage [58], while not interfering with attack maneuvers that involve only low predator displacement (small attack range). A possible explanation therefore is a shift in foraging tactic. After some days of starvation, scorpionfish may have started to actively search for prey [59], and consequently, prey could have benefited from hide/escape possibilities offered by habitat structure in the shrub habitat and (even more so) in the forest, as was the case against comber.

### 4.5. Vertical Movements in Structured Habitats in the Face of Multiple Predators

In the field, where wrasse co-exist with multiple predators, the wrasse anti-predator behavior when exposed to scorpionfish (i.e., increased occupancy of the open-water strata) is likely to increase wrasse predation risk from comber, other stalk-and-attack predators (e.g., *Serranus scriba*) and transient roving predators (e.g., *Dentex dentex*) [23]. Conversely, wrasse anti-predator behavior against active searchers, (i.e., seeking shelter within the vegetated strata), increases predation risk from sit-and-wait predators ambushing below the canopy. However, in the present study, putative interactive effects of multiple predators [26] were not tested. Wrasse were not exposed to both predators simultaneously since this would not provide reliable results considering the size of our experimental tanks. Nevertheless, our results suggest that the vertical stratification of the forest allows prey fish to adapt their anti-predator behavior very quickly by switching vertical strata, i.e., seeking vegetated strata versus avoidance, depending on the strategy of the predator. From this perspective, the interface canopy/open water in the forest could be seen as an ecotone where edge effects result in prey vertical movements, similarly to the ecotone between structured and unstructured habitats, where edge effects result in prey horizontal movements [28]. The adaptive shift of vertical strata involves only short-distance movements, and may allow wrasse to reduce their flight initial distance [60]. The lowest predator–prey distance being observed in the forest may support this hypothesis. Flight is costly in terms of both energy and loss of foraging opportunities. The immediate proximity of both vegetated and open-water strata in the forest may therefore have also positive side-effects with regards to the wrasse’s energy budget.

Although 3D movements related to anti-predator behaviors have rarely been documented, we believe they are common for many prey animals living in structured habitats. For instance, in a terrestrial system, Makin et al. [61] studied anti-predator behaviors of vervet monkeys (*Chlorocebus aethiops*), an African primate spending, in the savanna, an equal amount of time on the ground and in trees. The study highlighted an adaptive anti-predator behavior against multiple predators: when threatened by terrestrial predators (e.g., the leopard *Panthera pardus*) vervet monkeys can reduce predation risk by moving upwards into trees, while when threatened by aerial predators (e.g., eagles), they move down.

## 5. Conclusions

Our study highlighted that the habitat structure of the *Cystoseira* forest, including vertical stratification, may mediate lethal and behavioral predator–prey interactions that occur in 3D, and consequently may contribute to the co-existence of prey and multiple predators at higher densities in forest. To gain further insight into the mechanisms underlying the co-existence of prey and multiple predators within structured habitats, such as *Cystoseira* forests, kelp forests, seagrass meadows and mangrove roots, future studies should include 3D behavioral predator–prey interactions, and specific treatments for testing the putative interactive effects of multiple predators on prey anti-predator behavior. It would also be beneficial to carry out further experiments with the aim of assessing the relative contribution of differential mortality and habitat selection in shaping distribution patterns among habitats. Filling these gaps would help to predict fish assemblage structures under scenarios of greater human-driven losses of vegetated habitats, when habitat choice would no longer be possible.

## Figures and Tables

**Table 1:** PERMANOVA on Selection Index. For each species (the prey wrasse, the predator scorpionfish, the predator comber), comparison of the Selection Index (SI) between habitat treatment (Ha, 3 levels: Control Forest, Control Shrub and Choice Arena) and predator–prey treatments (Pr, 3 levels for the prey: the prey alone, the prey and the scorpionfish, the prey and the comber; 2 levels for each predator: the predator alone vs. the predator and the prey). ^ns^ not significant; ° *p* < 0.1; * *p* < 0.05; *** *p* < 0.001. See also Figure 3.

	Wrasse				Scorpionfish				Comber			
Source	df	SS	F		df	SS	F		df	SS	F	
Pr	2	0.68	1.38	^ns^	1	0.28	0.5	^ns^	1	0.13	0.86	^ns^
Ha	2	1.36	2.75	°	2	4.77	4.32	*	2	3.18	10.25	***
PrxHa	4	3.17	3.21	*	2	0.89	0.81	^ns^	2	0.09	0.3	^ns^
Res	34	8.4			18	9.94			25	3.87		
Total	42	14.65			23	15.87			30	7.72		

**Table 2:** PERMANOVA on proportions per activity category. For each species (the prey wrasse, the predator scorpionfish, the predator comber), comparison of its activity (proportions of motionless, hidden and moving) between habitat treatment (Ha, 3 levels: Forest (F), Shrub (S), Barren (B)) and predator–prey treatments (Pr, 3 levels for the prey: the prey alone (W), the prey and the scorpionfish (SW), the prey and the comber (CW); 2 levels for each predator: the predator alone vs. the predator and the prey). ^ns^ not significant; * *p* < 0.05; *** *p* < 0.001. Pair-wise comparisons are indicated in last row.

	Wrasse				Scorpionfish				Comber			
Source	df	SS	F		df	SS	F		df	SS	F	
Pr	2	6.03	129.74	***	1	0.02	4.66	*	1	0.34	111.23	***
Ha	2	0.05	1.13	^ns^	2	0.03	3.96	*	2	0.01	1.26	^ns^
PrxHa	3	0.03	0.37	^ns^	2	0.02	2.67	^ns^	1	0.00	0.01	^ns^
Res	28	0.65			18	0.06			19	0.06		
Total	35	7.87			23	0.12			23	0.47		
	Pr: W = SW ≠ CW				Ha: B = S; S = F; B ≠ F							

**Table 3:** PERMANOVA on mean and variation of fish individual’s vertical distribution. For each species (the prey wrasse, the predator scorpionfish, the predator comber), comparison of mean vertical position and variation in vertical position between habitat treatment (Ha, 3 levels: Forest (F), Shrub (S), Barren (B)) and predator–prey treatments (Pr, 3 levels for the prey: the prey alone (W), the prey and the scorpionfish (SW), the prey and the comber (CW); 2 levels for each predator: the predator alone vs. the predator and the prey). ^ns^ not significant; * *p* < 0.05; ** *p* < 0.01; *** *p* < 0.001. Results of pair-wise comparisons are indicated in Figure 4A.

			Mean Vertical Distribution			Variation in Vertical Distribution		
	Source	df	SS	F		SS	F	
Wrasse	Pr	2	283.59	57.70	***	22.56	9.44	***
	Ha	2	297.46	60.52	***	147.21	61.58	***
	Pr × Ha	3	109.74	14.89	***	8.18	2.28	^ns^
	Res	28	68.81			33.47		
	Total	35	960.22			288.36		
Scorpionfish	Pr	1	6.89	5.54	*	6.80	6.59	*
	Ha	2	14.31	5.75	**	15.36	7.44	**
	Pr × Ha	2	15.37	6.17	**	18.88	9.14	**
	Res	18	22.39			18.58		
	Total	23	58.95			59.61		
Comber	Pr	1	25.87	26.67	***	1.29	2.90	^ns^
	Ha	2	215.12	110.91	***	81.71	91.98	***
	Pr × Ha	1	0.68216	0.70	^ns^	0.05	0.11	^ns^
	Res	19	18.43			8.44		
	Total	23	241.27			94.93		

## Data Availability

All relevant data are within the paper and its Supporting Information files.

## Supplementary Materials

The following are available online at https://www.mdpi.com/article/10.3390/ani12070826/s1, Figure S1: Three habitat types in North-Western Mediterranean subtidal rocky reefs, Figure S2: Schematic representations of the observer position during behavioral observations, Figure S3: Pictures of artificial algae and experimental set up, Figure S4: Predator–prey distances depending on habitat-types and predator–prey treatments, Supplementary File S1 (Text and Figure S5): Method used for analyzing within-habitat short-term behavioral interactions and raw results. References [62,63] are citied in the Supplementary Materials.

## References

[1] Tews J., Brose U., Grimm V., Tielbörger K., Wichmann M.C., Schwager M., Jeltsch F. (2004). Animal Species Diversity Driven by Habitat Heterogeneity/Diversity: The Importance of Keystone Structures. J. Biogeogr..

[2] Gorman O.T., Karr J.R. (1978). Habitat Structure and Stream Fish Communities. Ecology.

[3] Alvarez-Filip L., Gill J.A., Dulvy N.K. (2011). Complex Reef Architecture Supports More Small-Bodied Fishes and Longer Food Chains on Caribbean Reefs. Ecosphere.

[4] Bostrom C., Jackson E.L., Simenstad C.A. (2006). Seagrass Landscapes and Their Effects on Associated Fauna: A Review. Estuar. Coast. Shelf Sci..

[5] Guidetti P. (2000). Differences Among Fish Assemblages Associated with Nearshore Posidonia Oceanica Seagrass Beds, Rocky–Algal Reefs and Unvegetated Sand Habitats in the Adriatic Sea. Estuar. Coast. Shelf Sci..

[6] Horinouchi M., Tongnunui P., Nanjyo K., Nakamura Y., Sano M., Ogawa H. (2009). Differences in Fish Assemblage Structures between Fragmented and Continuous Seagrass Beds in Trang, Southern Thailand. Fish. Sci..

[7] Schultz S., Kruschel C. (2010). Frequency and Success of Ambush and Chase Predation in Fish Assemblages Associated with Seagrass and Bare Sediment in an Adriatic Lagoon. Hydrobiologia.

[8] Schultz S.T., Kruschel C., Bakran-Petricioli T. (2009). Influence of Seagrass Meadows on Predator-Prey Habitat Segregation in an Adriatic Lagoon. Mar. Ecol.-Prog. Ser..

[9] Laegdsgaard P., Johnson C. (2001). Why Do Juvenile Fish Utilise Mangrove Habitats?. J. Exp. Mar. Biol. Ecol..

[10] Manson F.J., Loneragan N.R., Skilleter G.A., Phinn S.R. (2005). An Evaluation of the Evidence for Linkages between Mangroves and Fisheries. Oceanography and Marine Biology.

[11] Nanjo K., Nakamura Y., Horinouchi M., Kohno H., Sano M. (2011). Predation Risks for Juvenile Fishes in a Mangrove Estuary: A Comparison of Vegetated and Unvegetated Microhabitats by Tethering Experiments. J. Exp. Mar. Biol. Ecol..

[12] Anderson M.J., Millar R.B. (2004). Spatial Variation and Effects of Habitat on Temperate Reef Fish Assemblages in Northeastern New Zealand. J. Exp. Mar. Biol. Ecol..

[13] Jones G.P. (1984). Population Ecology of the Temperate Reef Fish Pseudolabrus Celidotus Bloch & Schneider (Pisces: Labridae). I. Factors Influencing Recruitment. J. Exp. Mar. Biol. Ecol..

[14] Cheminée A., Sala E., Pastor J., Bodilis P., Thiriet P., Mangialajo L., Cottalorda J.-M., Francour P. (2013). Nursery Value of *Cystoseira* Forests for Mediterranean Rocky Reef Fishes. J. Exp. Mar. Biol. Ecol..

[15] Bonaca M.O., Lipej L. (2005). Factors Affecting Habitat Occupancy of Fish Assemblage in the Gulf of Trieste (Northern Adriatic Sea). Mar. Ecol..

[16] Sala E., Ballesteros E., Dendrinos P., Di Franco A., Ferretti F., Foley D., Fraschetti S., Friedlander A., Garrabou J., Güçlüsoy H. (2012). The Structure of Mediterranean Rocky Reef Ecosystems across Environmental and Human Gradients, and Conservation Implications. PLoS ONE.

[17] Thiriet P.D., Di Franco A., Cheminée A., Guidetti P., Bianchimani O., Basthard-Bogain S., Cottalorda J.-M., Arceo H., Moranta J., Lejeune P. (2016). Abundance and Diversity of Crypto-and Necto-Benthic Coastal Fish Are Higher in Marine Forests than in Structurally Less Complex Macroalgal Assemblages. PLoS ONE.

[18] Cheminée A., Pastor J., Bianchimani O., Thiriet P., Sala E., Cottalorda J.-M., Dominici J.-M., Lejeune P., Francour P. (2017). Juvenile Fish Assemblages in Temperate Rocky Reefs Are Shaped by the Presence of Macro-Algae Canopy and Its Three-Dimensional Structure. Sci. Rep..

[19] Gotceitas V., Colgan P. (1989). Predator Foraging Success and Habitat Complexity: Quantitative Test of the Threshold Hypothesis. Oecologia.

[20] Hindell J.S., Jenkins G.P., Keough M.J. (2000). Evaluating the Impact of Predation by Fish on the Assemblage Structure of Fishes Associated with Seagrass (Heterozostera Tasmanica) (Martens Ex Ascherson) Den Hartog, and Unvegetated Sand Habitats. J. Exp. Mar. Biol. Ecol..

[21] Scharf F.S., Manderson J.P., Fabrizio M.C. (2006). The Effects of Seafloor Habitat Complexity on Survival of Juvenile Fishes: Species-Specific Interactions with Structural Refuge. J. Exp. Mar. Biol. Ecol..

[22] Nanjo K., Kohno H., Nakamura Y., Horinouchi M., Sano M. (2014). Effects of Mangrove Structure on Fish Distribution Patterns and Predation Risks. J. Exp. Mar. Biol. Ecol..

[23] Thiriet P., Cheminée A., Mangialajo L., Francour P. (2014). How 3D Complexity of Macrophyte-Formed Habitats Affect the Processes Structuring Fish Assemblages within Coastal Temperate Seascapes?. Underwater Seascapes.

[24] Sheaves M. (2001). Are There Really Few Piscivorous Fishes in Shallow Estuarine Habitats?. Mar. Ecol. Prog. Ser..

[25] Eklov P., Persson L. (1996). The Response of Prey to the Risk of Predation: Proximate Cues for Refuging Juvenile Fish. Anim. Behav..

[26] Martin C.W., Fodrie F.J., Heck K.L., Mattila J. (2010). Differential Habitat Use and Antipredator Response of Juvenile Roach (Rutilus Rutilus) to Olfactory and Visual Cues from Multiple Predators. Oecologia.

[27] Savino J., Stein R. (1989). Behavior of Fish Predators and Their Prey: Habitat Choice between Open Water and Dense Vegetation. Environ. Biol. Fishes.

[28] Smith T.M., Hindell J.S., Jenkins G.P., Connolly R.M., Keough M.J. (1989). Edge Effects in Patchy Seagrass Landscapes: The Role of Predation in Determining Fish Distribution. J. Exp. Mar. Biol. Ecol..

[29] Laurel B.J., Brown J.A. (2006). Influence of Cruising and Ambush Predators on 3-Dimensional Habitat Use in Age 0 Juvenile Atlantic Cod *Gadus Morhua*. J. Exp. Mar. Biol. Ecol..

[30] McCauley D., Micheli F., Young H., Tittensor D., Brumbaugh D., Madin E.P., Holmes K., Smith J., Lotze H., DeSalles P. (2010). Acute Effects of Removing Large Fish from a Near-Pristine Coral Reef. Mar. Biol..

[31] Gianni F., Bartolini F., Airoldi L., Ballesteros E., Francour P., Guidetti P., Meinesz A., Thibaut T., Mangialajo L. (2013). Conservation and Restoration of Marine Forests in the Mediterranean Sea and the Potential Role of Marine Protected Areas. Adv. Oceanogr. Limnol..

[32] Stergiou K.I., Karpouzi V.S. (2002). Feeding Habits and Trophic Levels of Mediterranean Fish. Rev. Fish Biol. Fish..

[33] Thiriet P. (2014). Comparisons of Fish Assemblage Structure and Underlying Ecologigal Processes, between *Cystoseira* Forests and Less Structurally Complex Habitats, in North-Western Mediterranean Subtidal Rocky Reefs. Ph.D. Thesis.

[34] Clarisse S. (1984). Apport de Différentes Techniques Cartographiques à La Connaissance de l’autoécologie de Cystoseira Balearica Sauvageau, Macroalgue Marine Dominante Dans La Région de Calvi (Corse).

[35] D’Anna G., Giacalone V.M., Fernandez T.V., Vaccaro A.M., Pipitone C., Mirto S., Mazzola S., Badalamenti F. (2012). Effects of Predator and Shelter Conditioning on Hatchery-Reared White Seabream *Diplodus sargus* (L., 1758) Released at Sea. Aquaculture.

[36] Fay M.P., Shaw P.A. (2010). Exact and Asymptotic Weighted Logrank Tests for Interval Censored Data: The interval R Package. J. Stat. Softw..

[37] R Development Core Team R (2013). A Language and Environment for Statistical Computing.

[38] Anderson M.J., Gorley R.N., Clarke K.R. (2008). PERMANOVA+ for PRIMER: Guide to Software and Statistical Methods.

[39] Jacobs J. (1974). Quantitative Measurement of Food Selection: A Modification of the Forage Ratio and Ivlev’s Electivity Index. Oecologia.

[40] Lima S.L. (1992). Strong Preferences for Apparently Dangerous Habitats—A Consequence of Differential Escape from Predators. Oikos.

[41] Gozler A.M., Kopuz U., Agirbas E. (2010). Seasonal Changes of Invertebrate Fauna Associated with Cystoseira Barbata Facies of Southeastern Black Sea Coast. Afr. J. Biotechnol..

[42] Lannin R., Hovel K. (2011). Variable Prey Density Modifies the Effects of Seagrass Habitat Structure on Predator−prey Interactions. Mar. Ecol. Prog. Ser..

[43] Horinouchi M., Mizuno N., Jo Y., Fujita M., Suzuki Y., Aranishi F., Sano M. (2013). Habitat Preference Rather than Predation Risk Determines the Distribution Patterns of Filefish Rudarius Ercodes in and around Seagrass Habitats. Mar. Ecol. Prog. Ser..

[44] Tait K.J., Hovel K.A. (2012). Do Predation Risk and Food Availability Modify Prey and Mesopredator Microhabitat Selection in Eelgrass (Zostera Marina) Habitat?. J. Exp. Mar. Biol. Ecol..

[45] Bell J.D., Westoby M. (1986). Abundance of Macrofauna in Dense Seagrass Is Due to Habitat Preference, Not Predation. Oecologia.

[46] Dahlgren C.P., Eggleston D.B. (2000). Ecological Processes Underlying Ontogenetic Habitat Shifts in a Coral Reef Fish. Ecology.

[47] Morris D.W. (2003). Toward an Ecological Synthesis: A Case for Habitat Selection. Oecologia.

[48] Alos J., March D., Palmer M., Grau A., Morales-Nin B. (2011). Spatial and Temporal Patterns in *Serranus cabrilla* Habitat Use in the NW Mediterranean Revealed by Acoustic Telemetry. Mar. Ecol. Prog. Ser..

[49] Viladiu C., Vandewalle P., Osse J.W.M., Casinos A. (1999). Suction Feeding Strategies of Two Species of Mediterranean Serranidae (*Serranus cabrilla* and *Serranus scriba*). Neth. J. Zool..

[50] Horinouchi M. (2007). Review of the Effects of Within-Patch Scale Structural Complexity on Seagrass Fishes. J. Exp. Mar. Biol. Ecol..

[51] Main K.L. (1987). Predator Avoidance in Seagrass Meadows—Prey Behavior, Microhabitat Selection, and Cryptic Coloration. Ecology.

[52] McCormick M.I., Lonnstedt O.M. (2013). Degrading Habitats and the Effect of Topographic Complexity on Risk Assessment. Ecol. Evol..

[53] Huey R.B., Pianka E.R. (1981). Ecological Consequences of Foraging Mode. Ecology.

[54] Harmelin-Vivien M.L., Kaim-Malka R.A., Ledoyer M., Jacob-Abraham S.S. (1989). Food Partitioning among Scorpaenid Fishes in Mediterranean Seagrass Beds. J. Fish Biol..

[55] Bascinar N.S., Saglam H. (2009). Feeding Habits of Black Scorpionfish *Scorpaena porcus*, in the South-Eastern Black Sea. Turk. J. Fish. Aquat. Sci..

[56] Demirhan S.A., Can M.F. (2009). Age, Growth and Food Composition of *Scorpaena porcus* (Linnaeus, 1758) in the Southeastern Black Sea. J. Appl. Ichthyol..

[57] Horinouchi M., Mizuno N., Jo Y., Fujita M., Sano M., Suzuki Y. (2009). Seagrass Habitat Complexity Does Not Always Decrease Foraging Efficiencies of Piscivorous Fishes. Mar. Ecol.-Prog. Ser..

[58] Rilov G., Figueira W.F., Lyman S.J., Crowder L.B. (2007). Complex Habitats May Not Always Benefit Prey: Linking Visual Field with Reef Fish Behavior and Distribution. Mar. Ecol. Prog. Ser..

[59] Savino J.F., Stein R.A. (1989). Behavioral interactions between fish predators and their prey—Effects of plant-density. Anim. Behav..

[60] Dill L. (1990). Distance-to-Cover and the Escape Decisions of an African Cichlid Fish, Melanochromis Chipokae. Environ. Biol. Fishes.

[61] Makin D.F., Payne H.F.P., Kerley G.I.H., Shrader A.M. (2012). Foraging in a 3-D World: How Does Predation Risk Affect Space Use of Vervet Monkeys?. J. Mammal..

[62] Husson F., Josse J., Pagès J. (2010). Principal Component Methods-Hierarchical Clustering-Partitional Clustering: Why Would We Need to Choose for Visualizing Data?. http://factominer.free.fr/more/HCPC_husson_josse.pdf.

[63] Lê S., Josse J., Husson F. (2008). FactoMineR: An R Package for Multivariate Analysis. J. Stat. Softw..

