# Supplementary material for: Adaptive Vertical Positioning as Anti-Predator Behavior: The Case of a Prey Fish Cohabiting with Multiple Predatory Fish within Temperate Marine Algal Forests
Source: Animals (Basel). 2022 Mar 24;12(7):826. doi: 10.3390/ani12070826 (PMC8997029; doi:10.3390/ani12070826)
Supplement: Supplementary file 1 [file animals-12-00826-s001.zip › animals-1512065-supplementary/animals-1512065-supplementary figures S1-S4.pdf]

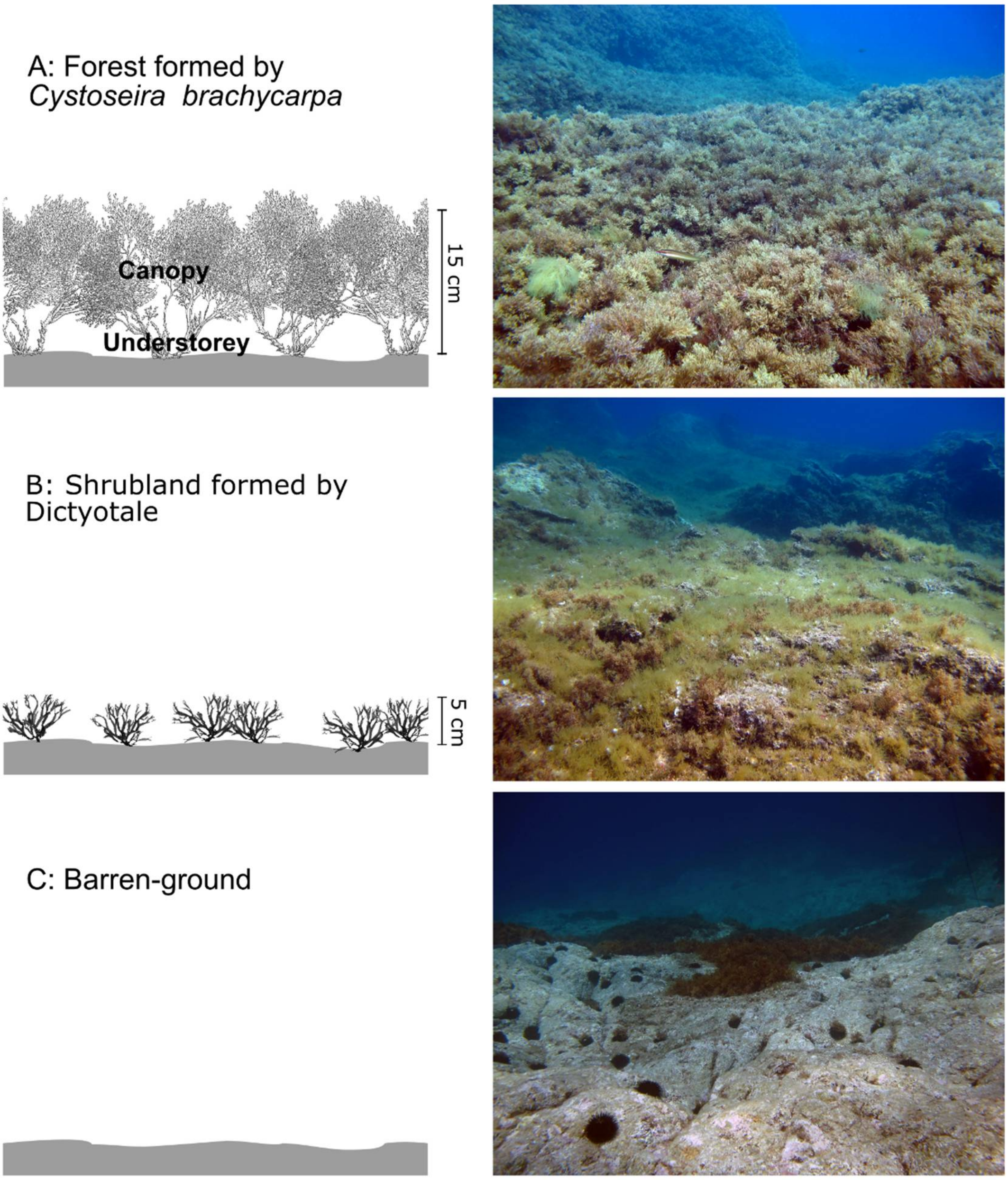

Supplementary material S1 (Figure S1). Three habitat types in North-Western Mediterranean subtidal rocky reefs. A: forest formed by the locally threatened species *Cystoseira brachycarpa* var. *balearica*, and B-C 2 habitat types that may replace lost *Cystoseira* forests; B: shrubs formed by Dictyotales and Sphacelariales; C: barren characterized by the absence of erect macrophytes. Left panel: schematic representations of the habitat structure provided by the dominant macrophytes. Habitat complexity decreases from A to C. Right panel: pictures taken in Corsica during summer 2011, at 8 m depth. Foregrounds span around 2 m width. Modified from Thiriet et al 2014 https://tel.archives-ouvertes.fr/tel-01083893

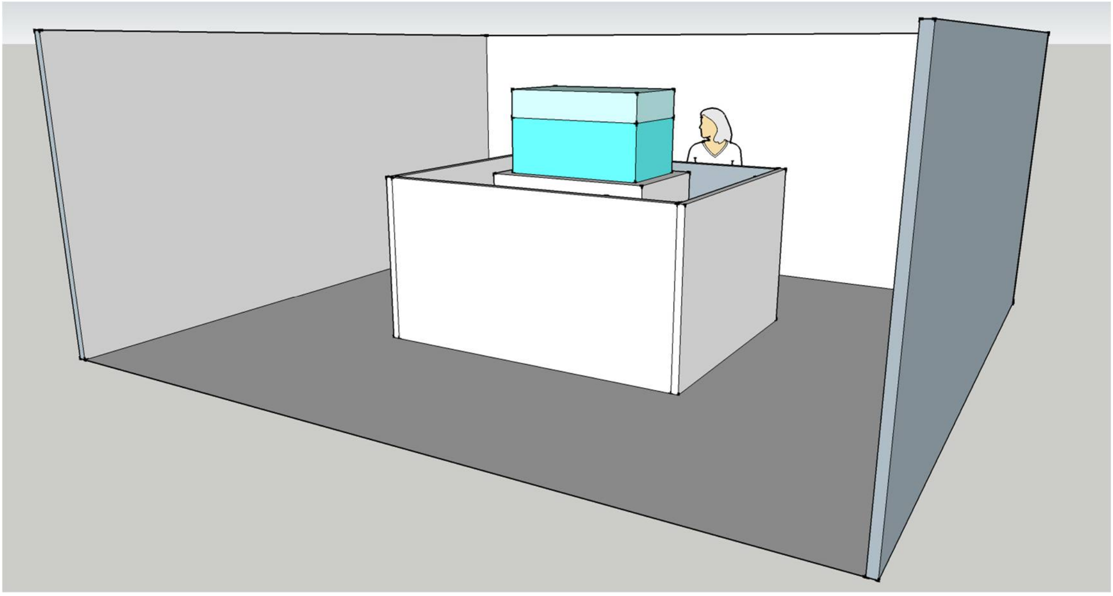

Supplementary material S2 (Figure S2). Schematic representations of the observer position during behavioral observations. The observer collected data by moving discretely all around the tank as often as necessary. The observer's body was hidden behind a 120 cm high wall that surrounded the tank at a distance of 60 cm from it. Moreover, tanks were isolated from each other by walls.

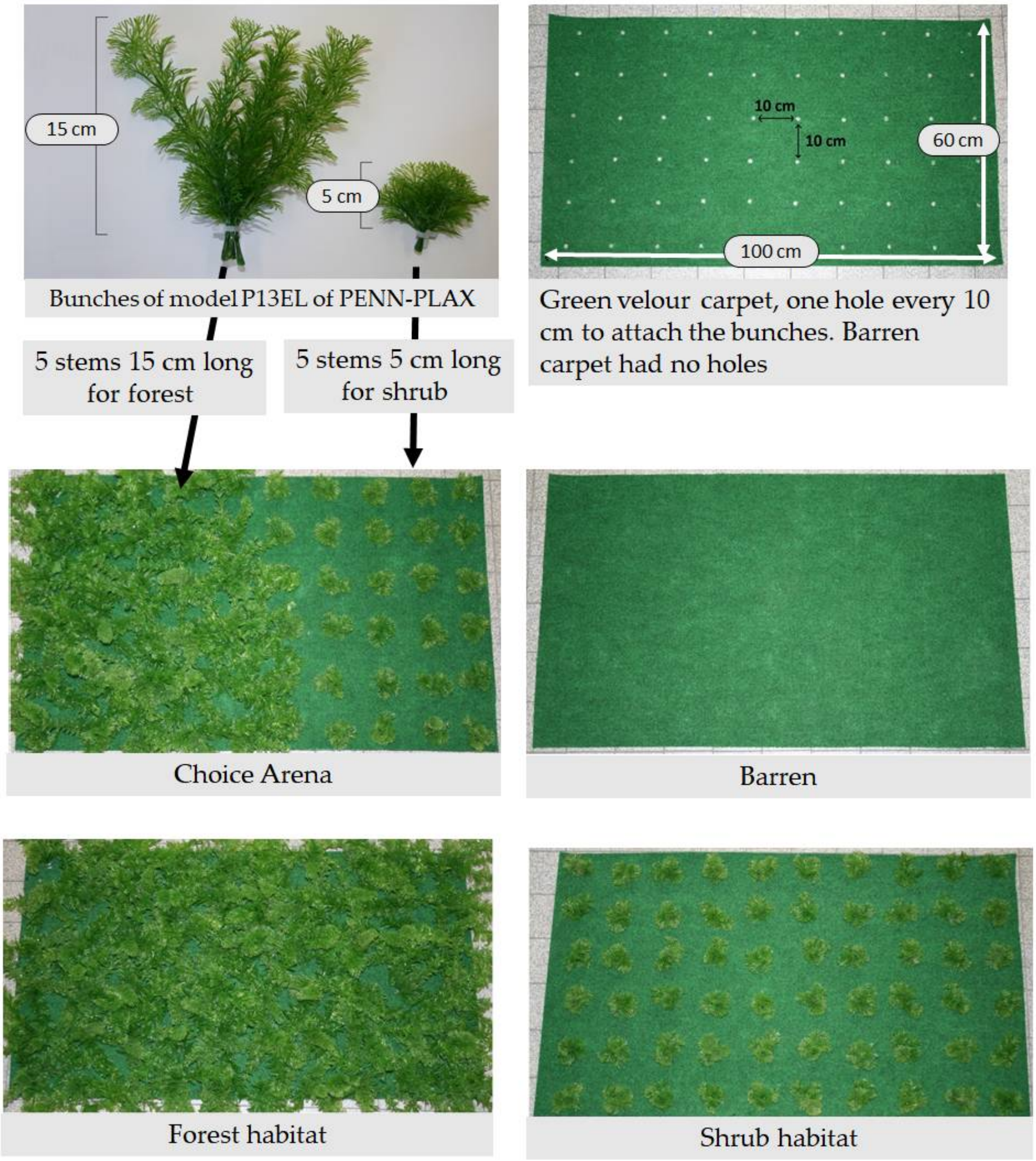

Supplementary material S3 (Figure S3). Pictures of artificial algae and artificial habitat used for tank experiments.

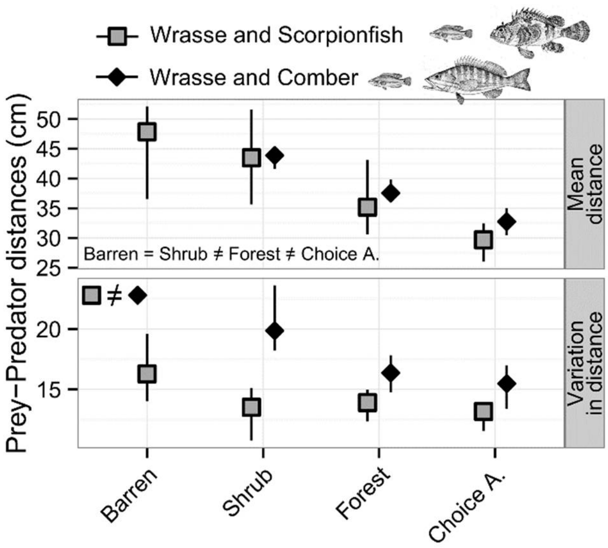

Supplementary material S4 (Figure S4). Prey-Predator distances depending on habitat-types and prey-predator treatments. Values averaged over replicates (95% CI) of the means (Mean distance) and the SDs (Variation in distance) of prey-predator distance distributions. Post-hoc to ANOVA, pair-wise comparison results are reported using equal/unequal signs

Supplementary Material File 1 (Text and Figure S5). Method used for analyzing within-habitat short-term behavioral interactions and raw results. For each prey-predator couple within each habitat, multivariate behavioral observations were clustered. The statistical unit was every single observation time. This allowed detection of tendencies between prey and predator respective positioning and activity, and their relative distance.
