# Supplementary material for: Adaptive Vertical Positioning as Anti-Predator Behavior: The Case of a Prey Fish Cohabiting with Multiple Predatory Fish within Temperate Marine Algal Forests
Source: Animals (Basel). 2022 Mar 24;12(7):826. doi: 10.3390/ani12070826 (PMC8997029; doi:10.3390/ani12070826)
Supplement: Supplementary file 1 [file animals-12-00826-s001.zip › animals-1512065-supplementary/animals-1512065-supplementary file S1-english.pdf]

Supplementary Material S5: Within-habitat description of short-term behavioral interactions

Statistical analyses

Within-habitat short-term prey–predator behavioral interactions were reconstructed from multiple-snapshot sampling by detecting tendencies between prey and predator respective positioning and activity, and their distance from each other. Separately for each combination of habitat (S, B, F) and prey–predator treatments (WS and WC), we carried out the three steps of the Husson et al. [1] method implemented in the R package 'FactoMineR' [2]. This enabled us to identify and characterize clusters of multivariate observations, which were interpretable as distinct phases of the short-term prey–predator behavioral interactions. First, we performed Multiple Correspondence Analysis (MCAs). Statistical units here were every single observational time, which were sampled every 30 sec within three sessions. The five categorical variables considered were: prey's Z position, predator's Z position, prey's activity, predator's activity, and PPD intervals. PPD was a continuous variable, so to enable possible non-linear relationships, we divided PPD into three or four balanced intervals depending on PPD distribution. For the categorical variables, we pooled case-by-case some categories for balancing distributions, since MCA is sensitive to imbalance. Secondly, we performed a partitioning of the observations’ coordinates on the principal components (PCs) using the HCPC routine (mixing hierarchical classification and K-means algorithms). We retained all the PCs needed to reach 85% of the cumulated explained variance (five to eight PCs) in order to stabilize the clustering by deleting the noise from the data [1]. The optimum number of clusters was assessed by selecting the partition (among 2 to 10 clusters) with the higher relative loss of inertia [1]. Thirdly, we identified which categories were characterizing the clusters by using the catdes routine, which computes and assesses significance (threshold used: 0.05) of the value test [2]. A significantly negative value test for a given category in a given cluster means that this category is under-represented in this cluster. A significant positive value test means over-representation. Hence, each cluster was characterized (positively or negatively) by some categories. This was interpretable as distinct phases of the short-term prey–predator interactions. The factor Trial (each couple of prey–predator individuals) was added as a supplementary categorical variable in order to detect consistent behavioral profiles from individual-specific behavioral profiles (the clusters that were characterized by some particular trial(s)).

Results

For every combination of prey–predator and habitat treatments, several behavioral profiles (i.e., clusters in MCA) were detected (Figure S4). None or few behavioral profiles were characterized by individual prey–predator couples (i.e., trial), suggesting that most of the behavioral profiles were not individual-specific but representative of the species

interactions. We thus report below only behavioral profiles that were not characterized by a trial.

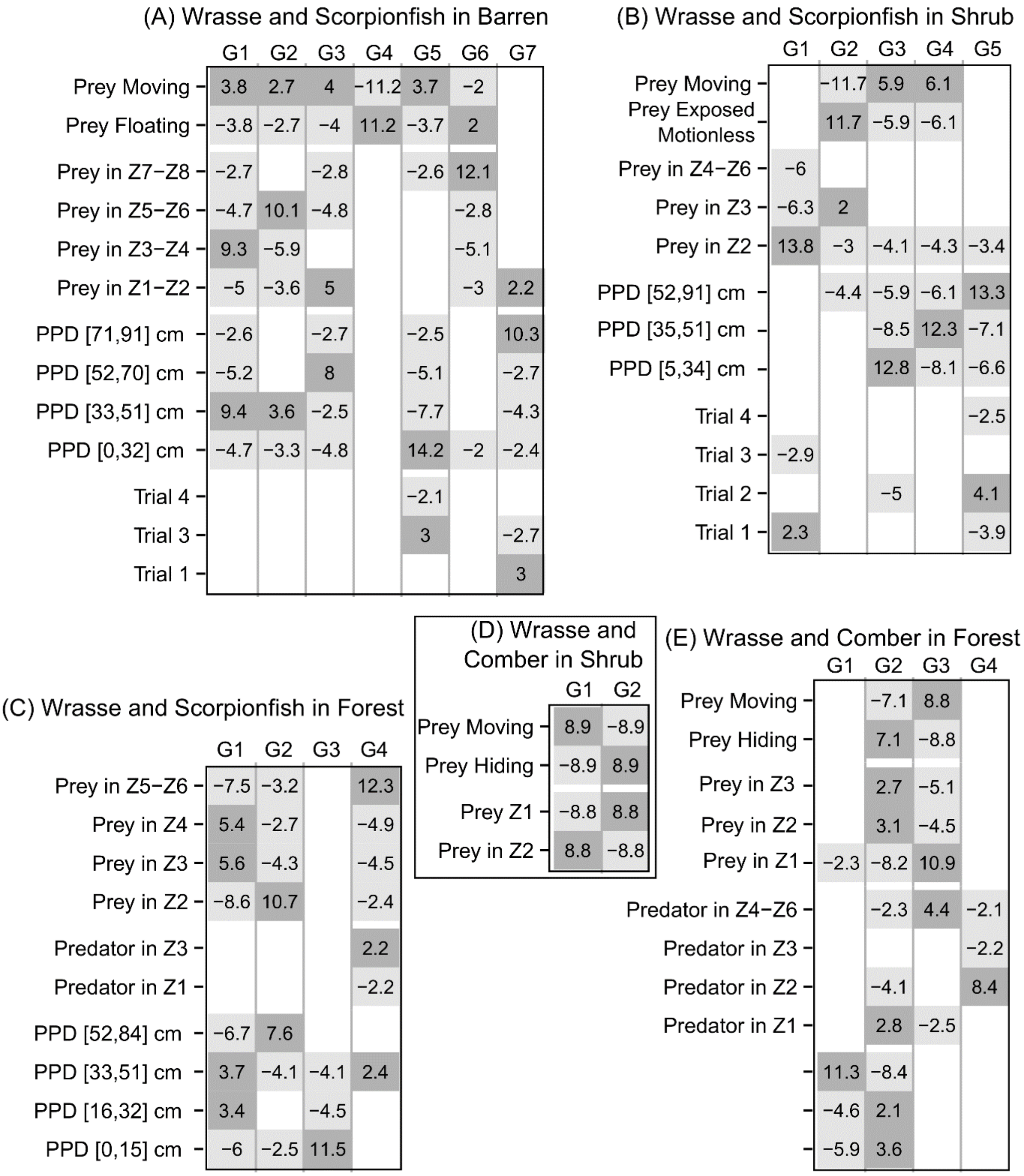

Figure S5. Value tests of the clustering used to detect tendencies between prey and predator respective positioning and activity, and their relative distance. For each prey–predator couple within each habitat (A to E), multivariate behavioral observations were clustered. To assess putative associations (positive or negative) among behavioral categories, a value test was carried out for each cluster (columns). Behavioral categories (rows) that were under-represented in the given cluster relative to the other clusters (value test significantly negative) are in light grey. Categories that were over-represented (value test significantly positive) are in dark grey. See also Figure 5.

Wrasse–scorpionfish behavioral interactions

Within the barren habitat, wrasse were moving in every strata of the water column except for the sub-surface (Z7-Z8). These movements were constrained since wrasse avoided scorpionfish by keeping a considerable distance from them (PPD $\in$ [52, 70] cm), especially when wrasse approached the bottom where scorpionfish were always lying in wait (Figure 5-A and Figure S4-A.G1.G2.G3). Sometimes, wrasse stopped moving to stay exposed motionless, with a preference for doing so in the sub-surface Z7-Z8 area (Figure S4-A.G4.G6).

Within the shrub, wrasse moved mainly 10 cm up to the shrubby strata and sometimes further (avoiding Z1 and Z2), keeping an intermediate distance from scorpionfish (PPD $\in$ [35, 51] cm), which were always lying within the shrubby strata (Figure 5-B and Figure S4-B.G4). Sometimes, wrasse stopped moving to stay exposed motionless, with a preference for doing so 10 cm up in the shrubby strata (Figure 5-B and Figure S4-B.G2).

Within the forest, wrasse were in the upper part of the canopy (Z3) and just above (Z4), while being at short-to-medium distances (PPD $\in$ [16, 51] cm) from scorpionfish lying on the bottom (Figure 5-C1 and Figure S4-C.G1). Wrasse were in the inner canopy (Z2) only when they were far (PPD $\in$ [52, 84] cm) from scorpionfish (Figure 5-C1 and Figure S4-C.G2). When scorpionfish were lying upon the canopy at the interface with open water (33% of observations), wrasse maintained intermediate distances (PPD $\in$ [33, 51] cm) by using the sub-surface (Figure 5-C2 and Figure S4-C.G4).

Wrasse–comber behavioral interactions

Within the shrub, wrasse were hidden motionless within the shrubby strata during 75% of our observations (Figure 5-D1 and Figure S4-D.G1). They sometimes moved, with a slight preference for moving just above the shrubby strata (15% of our observations) over moving around shrubs (9% of our observations) (Figure 5-D2 and Figure S4-D.G2).

Within the forest, wrasse were observed within the canopy (Z2 and Z3) in 86% of our observations, mostly hiding (75%) and sometimes moving (12%), while comber were at short-to-intermediate distances (PPD $\in$ [0, 44] cm), between the understory and above the canopy (Figure 6-E1 and Figure S4-E.G2). In contrast, wrasse only used the understory (Z1) for moving (12% of our observations) when comber were in or above the canopy (Figure 5-E2 and Figure S4-E.G3).

References

Husson F, Josse J, Pagès J (2010) Principal component methods - hierarchical clustering - partitional clustering: why would we need to choose for visualizing data?

Lê S, Josse J, Husson F (2008) FactoMineR: An R Package for Multivariate Analysis. Journal of Statistical Software 25:1-18
